# Complete mitochondrial genome of the endangered *Prunus pedunculata* (Prunoideae, Rosaceae) in China: characterization and phylogenetic analysis

**DOI:** 10.3389/fpls.2023.1266797

**Published:** 2023-12-08

**Authors:** Qian Liu, Zinian Wu, Chunyu Tian, Yanting Yang, Lemeng Liu, Yumei Feng, Zhiyong Li

**Affiliations:** ^1^ Institute of Grassland Research, Chinese Academy of Agricultural Sciences, Hohhot, China; ^2^ Key Laboratory of Grassland Resources and Utilization of Ministry of Agriculture, Hohhot, China

**Keywords:** *Prunus pedunculata*, endangered plants, mitochondrial genome, gene transfer, RNA editing, phylogenetic analysis

## Abstract

**Introduction:**

*Prunus pedunculata* (Prunoideae: Rosaceae), a relic shrub with strong resistance and multiple application values, is endangered in China. Extensive research had been devoted to gene expression, molecular markers, plastid genome analysis, and genetic background investigations of *P. pedunculata*. However, the mitochondrial genome of this species has not been systematically described, owing to the complexity of the plant mitogenome.

**Methods:**

In the present research, the complete mitochondrial genome of *P. pedunculata* was assembled, annotated, and characterized. The genomic features, gene content and repetitive sequences were analyzed. The genomic variation and phylogenetic analysis have been extensively enumerated.

**Results and discussion:**

The *P. pedunculata* mitogenome is a circular molecule with a total length of 405,855 bp and a GC content of 45.63%, which are the smallest size and highest GC content among the known *Prunus* mitochondrial genomes. The mitogenome of *P. pedunculata* encodes 62 genes, including 34 unique protein-coding genes (PCGs, excluding three possible pseudogenes), three ribosomal RNA genes, and 19 transfer RNA genes. The mitogenome is rich in repetitive sequences, counting 112 simple sequence repeats, 15 tandem repeats, and 50 interspersed repetitive sequences, with a total repeat length of 11,793 bp, accounting for 2.91% of the complete genome. Leucine (Leu) was a predominant amino acid in PCGs, with a frequency of 10.67%, whereas cysteine (Cys) and tryptophan (Trp) were the least adopted. The most frequently used codon was UUU (Phe), with a relative synonymous codon usage (RSCU) value of 1.12. Selective pressure was calculated based on 20 shared PCGs in the mitogenomes of the 32 species, most of which were subjected to purifying selection (Ka/Ks < 1), whereas *ccmC* and *ccmFn* underwent positive selection. A total of 262 potential RNA editing sites in 26 PCGs were identified. Furthermore, 56 chloroplast-derived fragments were ascertained in the mitogenome, ranging from 30 to 858 bp, and were mainly located across IGS (intergenic spacer) regions or rRNA genes. These findings verify the occurrence of intracellular gene transfer events from the chloroplast to the mitochondria. Furthermore, the phylogenetic relationship of *P. pedunculata* was supported by the mitogenome data of 30 other taxa of the Rosaceae family. Understanding the mitochondrial genome characteristics of *P. pedunculata* is of great importance to promote comprehension of its genetic background and this study provides a basis for the genetic breeding of *Prunus*.

## 1 Introduction

*Prunus pedunculata* Pall. (Prunoideae, Rosaceae), the longstalk almond, also known as *Amygdalus pedunculata* Pall., is a nationally endangered relic shrub (Ministry of Ecology and Environment of the People's Republic of China and Science, 2020; Yan et al., 2022) mainly distributed in the desert and mountain lands of arid and semi-arid regions in northwest China, Mongolia, and Russia (Wang et al., 2018c; He et al., 2021). Due to being constantly exposed to extreme climates in these regions, *P. pedunculata* has evolved great adaptability and resistance to water deficiency, low temperature, high wind, and barren soil, making it an optimal species for environmental restoration and sand fixation (Chu et al., 2013). *P. pedunculata* is an excellent oil-bearing plant and its seeds are rich in unsaturated fatty acids, which have high antihyperlipidemic and antioxidant activities (Gao et al., 2016). Longstalk almond nuts also contain health-promoting compounds, such as phytosterols, polyphenols, amygdalin (Chau and Wu, 2006), vitamins, minerals, and the essential amino acids (Gao et al., 2016; Wang et al., 2018b); thus, they possess great nutritional and medicinal value. *P. pedunculata* is also a valuable germplasm resource for wild fruit and feed plants (Wang et al., 2018c). Notwithstanding its multiple application values, *P. pedunculata* has become endangered due to extreme environmental conditions and anthropogenic activities, such as overexploitation, overgrazing, and environmental pollution (Chu et al., 2017). *P. pedunculata* has attracted significant attention ever since it was identified as a key protected wild plant (Class III) and endangered plant (Class II) of Inner Mongolia in the 1990s (Zhao, 1992; Chu et al., 2017). In addition, *P. pedunculata* was classified as a national near-threatened species by the China Biodiversity Red List – Higher Plants (Ministry of Ecology and Environment of the People's Republic of China and Science, 2020) and been assessed by the IUCN Red List of Threatened Species (Rhodes and Maxted, 2016). Extensive investigations have focused on chloroplast (cp) genome analysis (Duan et al., 2020; Du et al., 2021), chemical compounds (Ma, 2013; Lu et al., 2018; Yao et al., 2018; Li et al., 2020), genetic diversity (Zuo, 2016; Bao et al., 2021), resistance to various abiotic stress (Ma, 2006; Jiang, 2008; Luo, 2009; Guo, 2014), cultivation technology (Li, 2017; Wang et al., 2020b; Wang et al., 2021b), protection and utilization (Chu et al., 2015; Liu, 2017; Xiong et al., 2018; Bao et al., 2021) of *P. pedunculata*. Previous studies have indicated that *P. pedunculata* possesses many resistance genes. Understanding the genetic composition and phylogenetic status of *P. pedunculata* is of great academic value for its conservation and application.

Mitochondria, known as “the powerhouse of the cell”, are involved not only in adenosine triphosphate (ATP) synthesis through oxidative phosphorylation (Skippington et al., 2015), but also in programmed cell death, cell signaling, male sterility, and other angiosperm bioprocesses (McBride et al., 2006), and are semi-autonomous organelles found in most eukaryotic cells (Gualberto et al., 2014). Originated from endosymbiotic events of alpha-proteobacterial 1.5 billion years ago (Mower et al., 2012), the mitochondrial genome has evolved rapidly via multiple structural variation and rearrangements and gene transfers (Wu et al., 2020b). Plant mitogenomes vary in size, gene content, and genomic configuration compared with compact animal and fungal mitogenomes (Smith and Keeling, 2015; Morley and Nielsen, 2017). Besides, some unique characteristics exist in plant mitogenomes, including uncompact gene distribution, RNA editing, gene loss, DNA sequence transfer, and exogenous sequences acquisition (Knoop et al., 2011; Mower et al., 2012; Sloan et al., 2012). Plant mitochondrial genomes are very large and vary tremendously in size, even between close relatives (Kubo and Newton, 2008). Most mitogenomes range from 200-800 kb in length. The mitogenomes of spermatophytes are the greatest in size among all organelle genomes, which can be as large as 11.7 Mb in *Siberian larch* (Putintseva et al., 2020) and 11.3 Mb in *Silene Conica* (Sloan et al., 2012), whereas the smallest mitogenome by far is only 66 Kb, found in *Viscum scurruloideum* (Skippington et al., 2015). This tremendous variation in mitogenome size is assumed to be a consequence of repetitive sequences, DNA transfer from other organisms and large intragenic segments acquisition or loss (Bergthorsson et al., 2003; Wynn and Christensen, 2019; Wu et al., 2020b). Generally, plant mitochondrial genomes are circular double-linked DNA molecules (or circularly mapping molecules); such as single circular structure in *Arabidopsis thaliana* (Sloan et al., 2018b). In addition to the typical circular structure, branched, linear and multichromosomal architectures have also been observed in *Cucumis sativus*, *Oryza sativa*, *Silene noctiflora* (Bellot et al., 2016; Kazama and Toriyama; Kozik et al., 2019; Wu et al., 2020b), as well as an extreme example *Silene Conica*, which contains numerous circular chromosomes (Sloan et al., 2012; Wu et al., 2020b). Moreover, massive occurrence of gene transfer and RNA editing may lead to the gene content variation and sequence diversity of functional protein-coding genes (Rice et al., 2013; Wu et al., 2017; Sloan et al., 2018a). The complexity of mitogenome architectures due to genome recombination, duplication, and rearrangement makes the sequencing and assembly of mitogenomes much more complex and difficult than that of other organelle genomes (Fang et al., 2021). Hence, the full panoramic plant mitogenome description remains a bottleneck in evolutionary biology, and most plant phylogenetic studies have focused on nuclear and chloroplast genomes. Owing to the development of high-throughput sequencing technologies and the rising of next-generation phylogenomics, many software programs applicable to the mitogenome sequencing assembling were developed, such as GetOrganelle (Jin et al., 2020), Mitofiner (Allio et al., 2020), GSAT (He et al., 2023), and PMAT (https://github.com/bichangwei/PMAT), etc. The sequencing and assembly of mitogenome become much more accurately and efficiently.

To date (As of June 30, 2023), 895 complete plant mitogenomes have been deposited in the National Center for Biotechnology Information (NCBI) database (https://www.ncbi.nlm.nih.gov/), including 60 species of the Rosaceae family, among which 14 are *Prunus* species (including four cultivars). Most deposited mitogenomes maintain ‘master circle’ model (Wu et al., 2020b). Complete mitogenomes of the genus *Prunus* have been released in recent years (Pervaiz et al., 2015; Fang et al., 2021), however systematic studies have rarely been conducted. Phylogenetic relationship of *P. pedunculata* has been investigated based on morphology (Yazbek and Oh, 2013), microsatellite DNA (SSR) markers (Zhang et al., 2018), and molecular markers from the coding regions or non-coding regions of nuclear and chloroplast genes (Dong, 2015; Wang et al., 2020a), as well as chloroplast genomes (Wang et al., 2018a; Duan et al., 2020; Wang et al., 2020a). However, the phylogenetic affinities of *P. pedunculata* have not yet been determined from the perspective of the mitogenome. Elucidating the mitochondrial genome of *P. pedunculata* is a prerequisite for accurate molecular identification and genetic breeding of this endangered species. In this report, the mitogenome of this species was comprehensively assembled and analyzed. Genomic features, repetitive sequences, codon usage of PCGs, RNA editing sites, synonymous substitution rates, and DNA sequence transfer events in the *P. pedunculata* mitogenome have been extensively enumerated. Given the paucity of plant mitogenome information, the phylogenetic analysis was referring to available mitogenome data for only 30 previously annotated species of the Rosaceae family based on 20 conserved PCGs, which further clarify the evolutionary relationships and genetic background of *Prunus* species. Concurrently, the decipherment of the mitogenome enriches molecular markers and genetic resources for *Prunus* breeding and provides in-depth knowledge of organelle genome evolution.

## 2 Materials and methods

### 2.1 DNA extraction, genome sequencing, and assembly

Fresh leaves of *P. pedunculata* were collected from Hohhot, Inner Mongolia, China (40.57°N, 111.93°E) and deposited in the National Medium-Term Genebank Forage Germplasm (Hohhot, China). Genomic DNA was extracted from fresh leaves using a Plant DNA Isolation Kit (Tiangen, Beijing, China) and sequenced using an Illumina MiSeq platform (Novogene Co., Ltd., Tianjing, China). Around 7.52 Gb clean data with 50.16 million reads were yield and used for mitogenome *de novo* assembling. The assembly of mitochondria was performed using the software GetOrganelles V 1.7.5.3 (Jin et al., 2020) with default parameters (-R 50 -k 21,45,65,85,105,115,127 -P 1000000) (Jin et al., 2020). The accuracy of the assembly results was checked using the visualization software Bandage (Wick et al., 2015) and by mapping clean reads using Bowtie2 (Langmead and Salzberg, 2012). Afterwards, the average coverage depth was assessed to be 242.9x by SAMtools (Li et al., 2009) (**Supplementary Figure S1**). The coverage was visualized by Integrative Genomics Viewer (IGV) (Thorvaldsdottir et al., 2012). The cp genome of *P. pedunculata* was assembled in a similar manner. The complete mitochondrial genome sequence was deposited in GenBank (accession number: OQ 556854.1) and the complete chloroplast genome sequence was deposited with accession numbers of OR343251.

### 2.2 Genome annotation

*P. pedunculata* mitogenomes were annotated using GeSeq (Tillich et al., 2017) with reference to previously released mitogenome data of *Prunus* species and then manually adjusting the data into a circular mitogenome model. The cp genome was annotated using Plastid Genome Annotator (PGA) tools (Qu et al., 2019). Subsequently, Geneious V9.0.2 was used to amend mistaken codons (Kearse et al., 2012). The genome map was visualized using the Organellar Genome Draw (OGDRAW) software (Greiner et al., 2019).

### 2.3 Repeat sequence identification

Simple sequence repeats (SSRs) of the *P. pedunculata* mitochondrial genome were identified using the MISA software (Beier et al., 2017) with the parameters of minimum nucleotide numbers of mono-10, di-6, tri-4, tetra-3, penta-3 and hexa-3, respectively. Tandem Repeats Finder (TRF) (Benson, 1999) was used to identify tandem repeats with default parameters, whereas dispersed repeats larger than 70 bp were identified as forward, reverse, palindromic, and complementary repeats using the online tool REPuter (Kurtz et al., 2001) with a Hamming distance of 3 and a cutoff *e*-value of 1*e*-5.

### 2.4 Codon usage bias analysis

The RSCU value of PCGs and their amino acid composition were calculated by the Molecular Evolutionary Genetics Analysis software (MEGA v11.0.26) (Tamura et al., 2021), codon preferences were configured using Perl scripts.

### 2.5 Selective pressure calculation

Non-synonymous (Ka) and synonymous (Ks) substitution rates were calculated using DnaSP 6.12.0 (Rozas et al., 2017), based on a total of 20 shared PCGs (*atp1*, *atp4*, *atp6*, *atp8*, *atp9*, *ccmB*, *ccmC*, *ccmFc*, *ccmFn*, *cob*, *cox2*, *cox3*, *mttB*, *nad2*, *nad3*, *nad4*, *nad5*, *nad6*, *nad9*, and *rps13*) between the mitogenomes of *P. pedunculata* and 29 other Rosaceae species and *Oryza sativa*, *Triticum aestivum*.

### 2.6 Prediction of RNA editing sites

Based on three RNA-seq datasets of *P. pedunculata* deposited in the SRA database (https://www.ncbi.nlm.nih.gov/sra/; accession numbers: SRR13261917, SRR13261918 and SRR13261919) (Bao et al., 2021), we identified the putative RNA editing sites in mitochondrial PCGs. Mapping the RNA-seq data onto the sequences of mitochondrial PCGs by using BWA v0.7.15 (Li and Durbin, 2010) software. Then we called single nucleotide polymorphism sites (SNPs) by using SAMtools v1.17 (Li et al., 2009) and BCFtools v1.17 (Danecek et al., 2021). To identify and annotate RNA editing sites, the SNP-calling data were processed using REDO v 1.0 (Wu et al., 2018), a specialized tool for easily identifying RNA editing sites in plant organelles. To exclude the false positive RNA editing sites, BWA v0.7.15 was used to mapping the DNA-Seq data to *P. pedunculata* mitogenome. The SNP-calling method was conducted using BCFtools, then eliminating the RNA editing sites detected in genomic SNPs.

### 2.7 Chloroplast-derived mitochondrial sequence identification

The chloroplast genome data for *P. pedunculata* were obtained from our assemblies. Homologous sequences between the chloroplast genome and mitogenome were identified, and the transferred DNA fragments were screened using BLASTN with a cutoff value of 1*e*-5. Gene transfer from the chloroplasts to mitochondria was visualized using TB tools (Chen et al., 2020).

### 2.8 Phylogenetic analysis

Phylogenetic analysis based on 20 shared PCGs (as mentioned in Section 2.5) derived from the complete mitogenomes of 30 selected Rosaceae species was performed, with *O. sativa* and *T. aestivum* as the outgroups. Mitogenome data of the reference accessions were downloaded from the NCBI (**Supplementary Table S1**). The corresponding nucleotide sequences of the PCGs in the chosen genomes were concatenated, and the MAFFT program (Katoh and Standley, 2013) was used to perform multiple sequence alignment. Both the ML algorithm and Bayesian methods were used to construct the phylogenetic tree, and the best models were selected using ModelFinder (Kalyaanamoorthy et al., 2017). The ML method was conducted using RAxML (Stamatakis, 2006) with the GTRGAMMA model and bootstrap of 1000 replicates. Bayesian inferences (BI) using MrBayes v3.2.6. (Ronquist et al., 2012) were calculated to select the best-of-fit model GTR+F+I+G4.

## 3 Results

### 3.1 Genomic features of the *P. pedunculata* mitogenome

**Figure 1:**
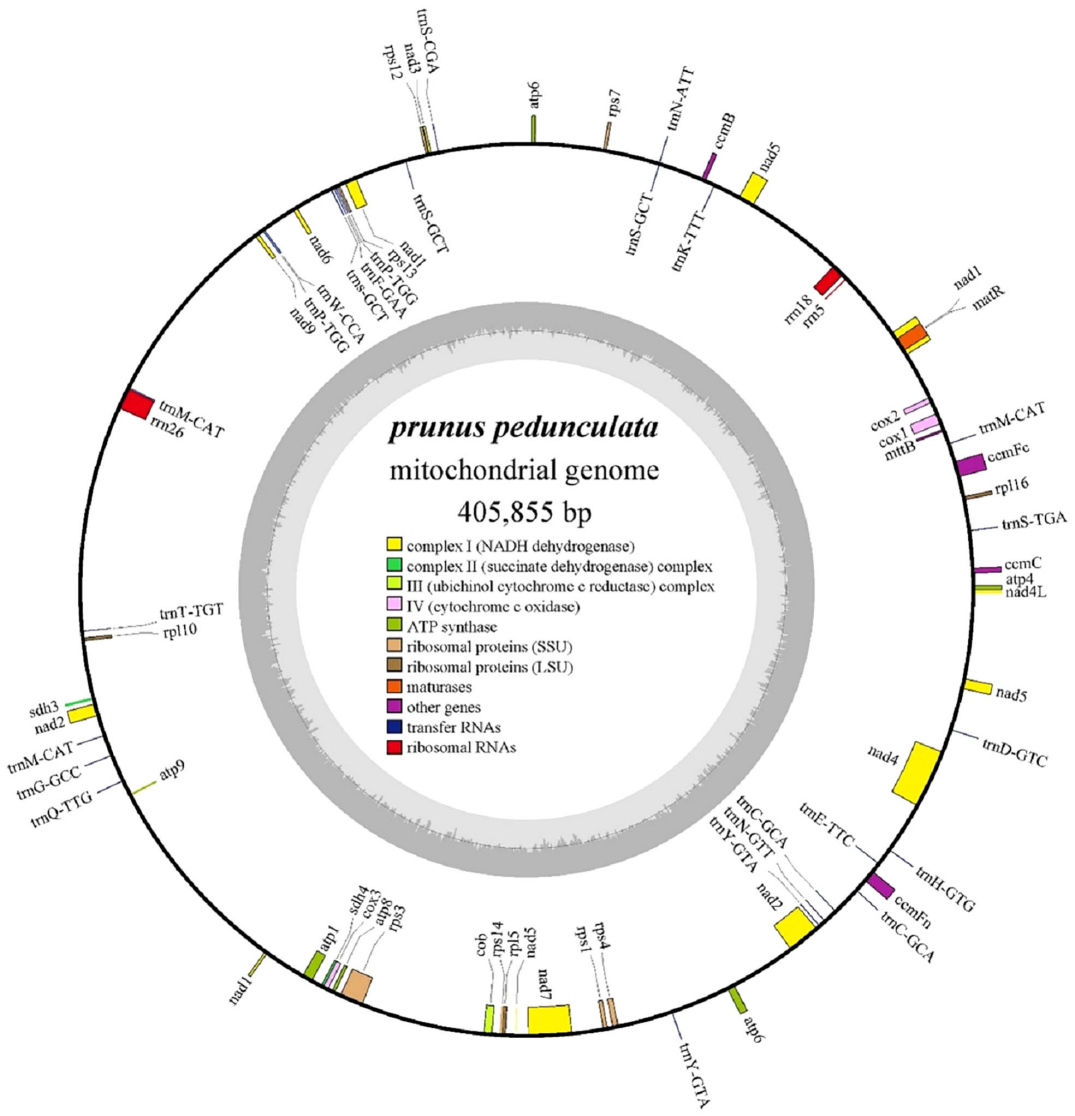
The circular map of the complete mitogenome of *P. pedunculata*. Genes located on the outside and inside of the circle were forward and reverse transcribed, respectively. The charcoal grey colored region in the inner circle depicts the GC content. Different functional gene groups are color coded.

The complete mitochondrial genome of *P. pedunculata* was assembled into a typical single circular molecule with a size of 405,855 bp (**Figure 1**) and a GC content of 45.63% (**Table 1**). The mitogenome comprised 27.06% adenine, 27.31% thymine, 22.77% guanine, and 22.85% cytosine. Although 84.82% of the mitogenome was composed of non-coding regions, the proportion of PCGs and cis-spliced-introns was 7.39% and 6.58% in the mitogenome, respectively, and that of tRNA and rRNA accounted for 0.45% and 1.27%, respectively.

**Table 1:** Genomic features of the *P. pedunculata* mitogenome.

Feature	A (%)	T (%)	G (%)	C (%)	GC (%)	Size (bp)	Proportion in Genome (%)
Genome	27.06	27.31	22.77	22.85	45.63	405855	100
Protein-coding genes	26.27	30.8	21.62	21.31	42.93	29973	7.39
Cis-spliced-intron	25.67	22.74	27.05	24.54	51.59	26711	6.58
tRNA	22.88	26.52	27.93	22.66	50.6	1840	0.45
rRNA	26.17	21.88	29.19	22.76	51.95	5136	1.27
Non-coding regions	27.34	27.34	22.75	22.57	45.32	344248	84.82

The mitochondrial genome of *P. pedunculata* contains 14 core genes, including five ATP synthase genes (*atp1, atp4, atp6, atp8, atp9*), four cytochrome C biogenesis genes (*ccmB, ccmC, ccmFc, ccmFn*), three cytochrome c oxidase genes (*cox1, cox2, cox3*), one ubiquinol cytochrome c reductase gene (*cob*), and one maturase gene (*matR*) (**Table 2**). In addition, an *atp6*-like pseudogene was detected. The genome also comprises 20 variable genes, three ribosomal RNAs (*rrn18, rrn26, rrn5*), and 19 transfer RNAs. These variable genes included nine NADH dehydrogenase genes (*nad1, nad2, nad3, nad4, nad4L, nad5, nad6, nad7, nad9*), two large ribosome protein subunits (*rpl10, rpl5)*, six small ribosome protein subunits (*rps1, rps12, rps13, rps3, rps4, rps7*), one transport membrane protein (*mttB*), and two succinate dehydrogenase genes (*sdh3, sdh4*). In addition, the *rpl16*-like and a *rps14*-like sequence*s* were identified as pseudogenes. As for transfer RNAs, *trnS-GCT* and *trnM-CAT* had three copies, while t*rnC-GCA*, *trnP-TGG*, and *trnY-GTA*, had two copies. Other tRNA genes were represented by identical copies in the mitogenome. Intron sequences were identified in nine genes (**Table 2**), among which *nad1*, *nad2*, *nad5*, and *nad7* contained four introns; *nad4* contained three introns; and *ccmFc*, *cox2*, *rps3*, and one copy of *trnY-GTA* possessed one intron region.

**Table 2:** Gene composition in the *P. pedunculata* mitogenome.

	Group of genes	Gene name
Core genes	ATP synthase	*#atp6, atp1, atp4, atp6, atp8, atp9*
	Cytochrome c biogenesis	ccmB, ccmC, ccmFc*, ccmFn
	Ubiquinol cytochrome c reductase	*cob*
	Cytochrome c oxidase	cox1, cox2^*^, cox3
	Maturases	*matR*
Variable genes	Transport membrane protein	*mttB*
	NADH dehydrogenase	nad1^****^, nad2^****^, nad3, nad4^***^, nad4L, nad5^****^, nad6, nad7^****^, nad9
	Ribosomal proteins (LSU)	*#rpl16, rpl10, rpl5*
	Ribosomal proteins (SSU)	#rps14, rps1, rps12, rps13, rps3*, rps4, rps7
	Succinate dehydrogenase	*sdh3, sdh4*
rRNA genes	Ribosomal RNAs	*rrn18, rrn26, rrn5*
tRNA genes	Transfer RNAs	trnC-GCA (2), trnD-GTC, trnE-TTC, trnF-GAA, trnG-GCC, trnH-GTG, trnK-TTT, trnM-CAT (3), trnN-ATT^*^, trnN-GTT, trnP-TGG (2), trnQ-TTG, trnS-CGA, trnS-GCT(3), trnS-TGA, trnT-TGT^*^, trnW-CCA, trnY-GTA, trnY-GTA^*^

Asterisks (^*^) beside genes represent intron numbers; Pound (#) before genes indicates Pseudogene; Numbers (2 or 3) after genes show the number of copies of multi-copy genes.

### 3.2 Anatomization of repeat sequences

**Figure 2:**
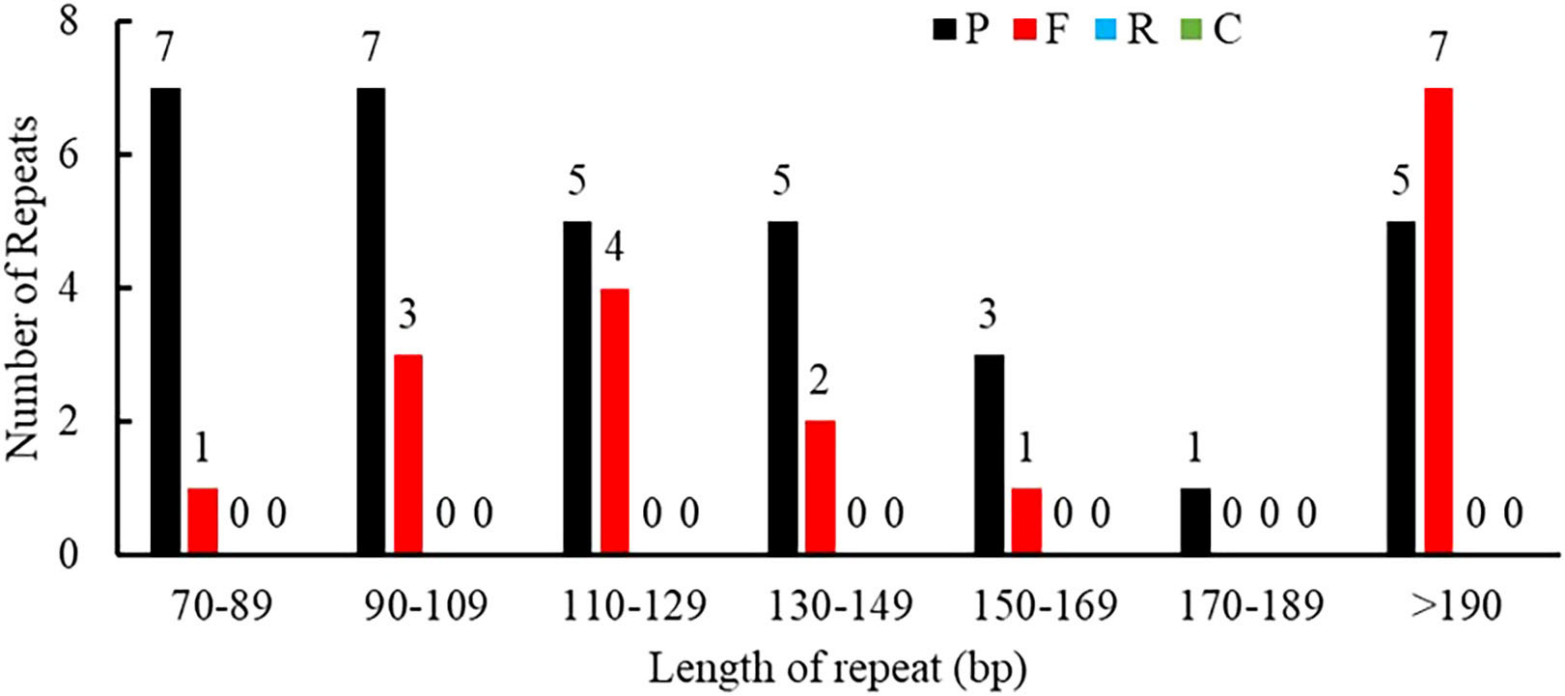
Allocation of the lengths of dispersed repeats in the *P. pedunculata* mitogenome. The X-axis indicates the types of dispersed repeats and the ordinate indicates the number of scattered repeats.

Repeat sequences included SSRs, tandem repeats, and dispersed repeats. A total of 50 interspersed repeats were identified in the *P. pedunculata* mitogenome, including 33 palindromic repeats (P, 66%) and 17 forward repeats (F, 34%); no reverse or complement repeats were detected. The lengths of the repeats were unevenly distributed. Most of the repeat sizes were between 70 bp-190 bp (78%), and 11 repeats (22%) exceeded 200bp, whereas only one repeat was longer than 1 kb, which was the largest repeat (1257 bp) (**Figure 2**; **Supplementary Table S2**).

**Figure 3:**
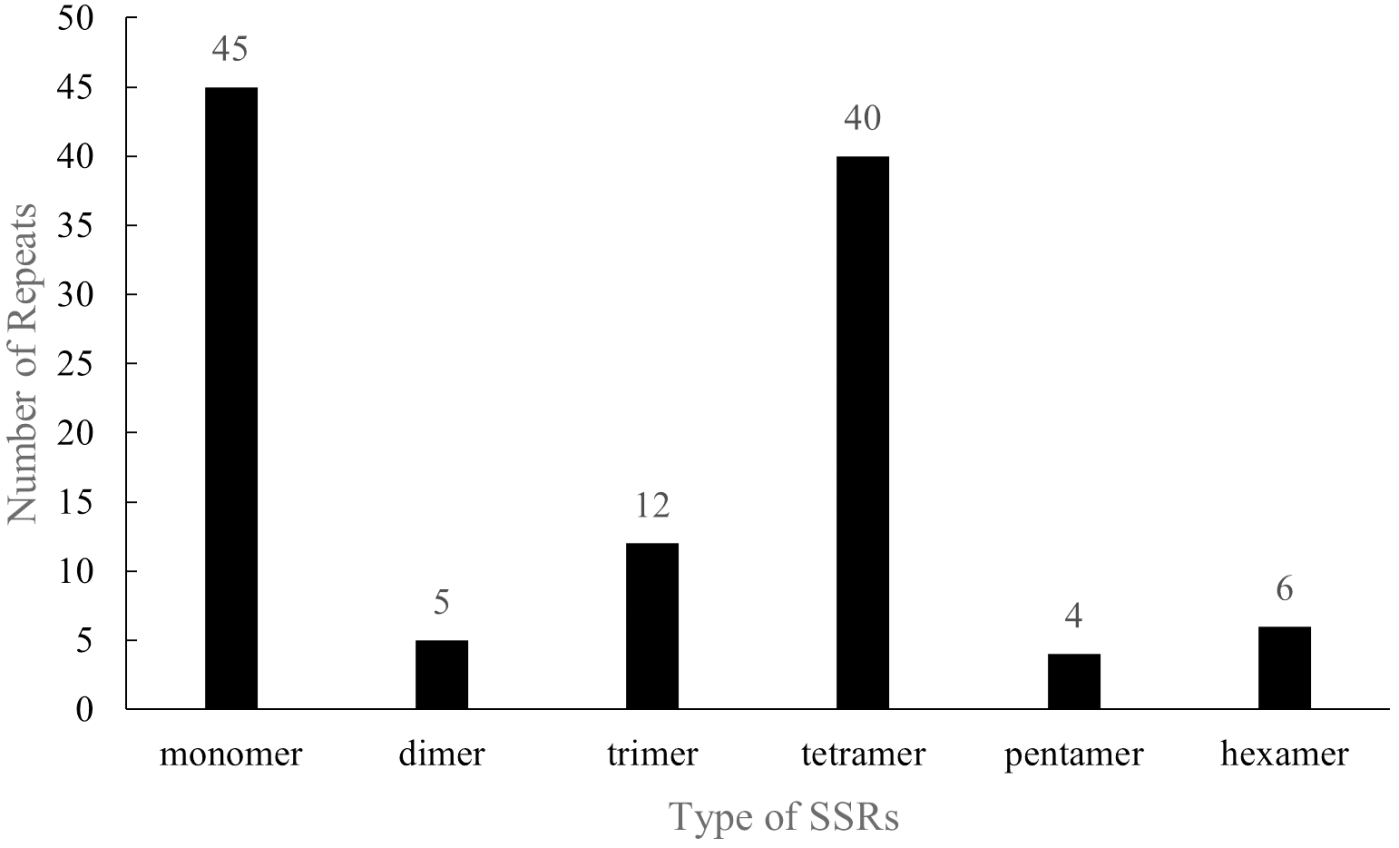
Distribution of SSRs in the *P. pedunculata* mitogenome. The X-axis indicates the type of SSRs, and the ordinate indicates the number of SSR repeats.

With regards to the SSRs, the distribution and structure of 112 SSR repeats in the *P. pedunculata* mitogenome were analyzed, comprised 45 single-nucleotide motifs (40.18%), five dinucleotide repeats (4.46%), 12 trinucleotide repeats (10.71%), 40 tetranucleotide repeats (35.71%), four pentanucleotide repeats (3.57%), and six hexanucleotide repeats (5.36%) (**Figure 3**; **Supplementary Table S3**). Among all the SSRs, most repeats (82, 72.32%) were rich in A/T. Notably, 34 SSRs were entirely composed of A/T, including 26 monomer units (A/T), three dimer units (AT/TA), two trimer units (AAT/TTA, TAT/ATA), and three tetramer units (AAAT/ATTT, AATT/TTAA). The A/T richness of the 48 SSRs ranged from 50 to 80%. The majority of SSRs (95) were located in IGS region, while nine SSRs were distributed on introns, two on exons, and one on the ORF region of *rps1*; only one SSR repeat was positioned across the intron region of *nad1* and part of the coding region of *matR* (**Supplementary Table S4**). These extensive SSRs provide abundant potential molecular markers for the identification and genetic study of *Prunus*.

Additionally, 15 tandem repeat sequences ranging from 14 to 51 bp were evenly distributed in the mitogenome of *P. pedunculata* with a similarity match greater than 80%. These tandem repeats were predominantly located in the IGS, and only one repeat resided on *rrn26* (**Supplementary Table S5**). However, not all the repeat sequences were copied. Certain sequences had multiple non-integral copies. For instance, the TACATATTCGAGAA motif was repeated twice in the IGS between *atp9* and *nad1*, whereas the GACTATGAAACAGATCGC repeat unit was present in *rrn26* with a repetition number of 2.4.

### 3.3 Codon usage analysis of PCGs

**Figure 4:**
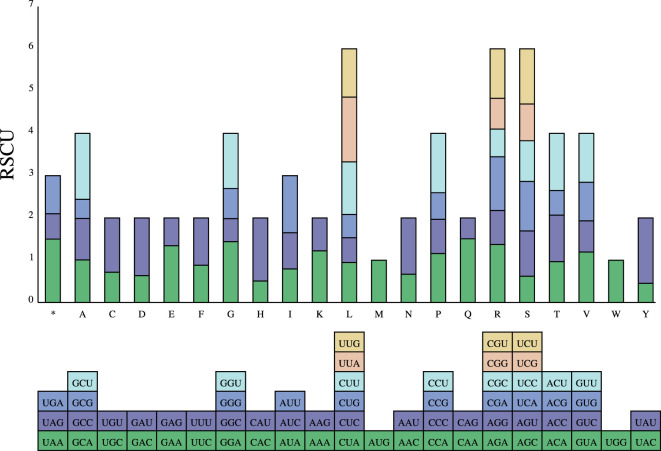
Relative synonymous codon usage (RSCU) in PCGs of *P. pedunculata* mitogenome. Codon families are on the abscissa.

The codon usage of 34 PCGs in the *P. pedunculata* mitogenome with a total length of 29,973 bp, encoding 9991 codons was analyzed. The results of RSCU analysis are shown in **Figure 4**. Leucine (Leu) was a predominant amino acid in PCGs with a frequency of 1066 (10.67%), followed by serine (8.98%) and isoleucine (7.97%), whereas cysteine (Cys) and tryptophan (Trp) were the least adopted amino acids, which only occurred 146 and 145 times (1.46% and 1.45%, respectively). The PCGs had the highest preference for UUU (Phe), which was used 364 times in PCGs, with an RSCU value of 1.12. The TAG termination codon was the least frequently used (**Table 3**; **Supplementary Table S6**). Alanine (Ala) had a preference for GCT, which occasionally occurred 248 times in PCGs, with a maximum RSCU value of 1.56.

**Table 3:** Codon counts in the *P. pedunculata* mitochondrial PCGs.

Codon	Count	Codon	Count	Codon	Count	Codon	Count
UAA(*)	15	GGC(G)	93	AUG(M)	271	AGU(S)	160
UAG(*)	6	GGG(G)	122	AAC(N)	110	UCA(S)	175
UGA(*)	9	GGU(G)	223	AAU(N)	219	UCC(S)	144
GCA(A)	160	CAC(H)	64	CCA(P)	160	UCG(S)	130
GCC(A)	156	CAU(H)	188	CCC(P)	112	UCU(S)	195
GCG(A)	72	AUA(I)	211	CCG(P)	87	ACA(T)	123
GCU(A)	248	AUC(I)	227	CCU(P)	194	ACC(T)	140
UGC(C)	52	AUU(I)	358	CAA(Q)	217	ACG(T)	74
UGU(C)	93	AAA(K)	237	CAG(Q)	71	ACU(T)	172
GAC(D)	104	AAG(K)	151	AGA(R)	149	GUA(V)	191
GAU(D)	223	CUA(L)	168	AGG(R)	87	GUC(V)	118
GAA(E)	284	CUC(L)	104	CGA(R)	138	GUG(V)	146
GAG(E)	139	CUG(L)	98	CGC(R)	71	GUU(V)	185
UUC(F)	287	CUU(L)	221	CGG(R)	79	UGG(W)	146
UUU(F)	364	UUA(L)	272	CGU(R)	127	UAC(Y)	70
GGA(G)	246	UUG(L)	203	AGC(S)	93	UAU(Y)	239

Interestingly, in the PCGs, A/T bases preferentially appeared in the third codon position, rather than C/G. Codons ending in A/T had RSCU values greater than 1. In addition, almost all PCGs began with the typical start codon ATG, except for *nad1* which started with ACG. Five models of termination codons were observed in the PCGs. TNAs (TAA, TGA, and N for A, T, C, or G, respectively) were the most dominant codons in the 24 PCGs. Six PCGs (*atp4*, *mttB*, *matR*, *nad7*, *rps1*, and *ccmFn*) were terminated with TAG. Meanwhile, *ccmFc*, *atp9*, and *sdh4* were stopped by CGA and *atp6* ended with CAA, which may be incomplete codons (**Supplementary Table S7**).

### 3.4 The substitution rates of mitochondrial PCGs

**Figure 5:**
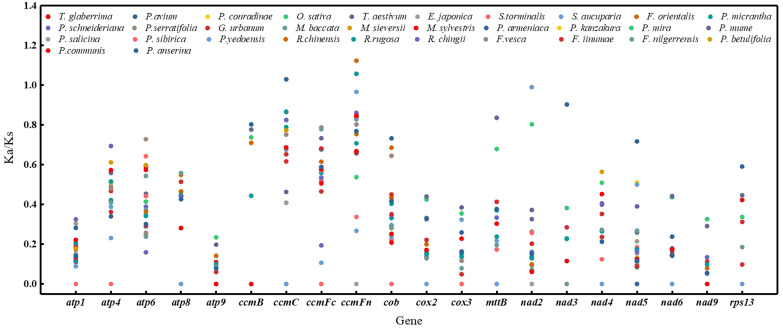
A dot plot of the Ka/Ks values of 20 protein-coding genes in mitogenomes of *P. pedunculata* versus 32 species.

Non-synonymous and synonymous substitution ratios (Ka/Ks) were calculated for the mitogenomes of 32 species based on 20 homologous PCGs. *P. pedunculata* was used as the reference. In most PCGs, the Ka/Ks values were notably less than 1 (**Figure 5**; **Supplementary Table S8**), implied that these genes were dominated by purifying selection during evolution. Conversely, the Ka/Ks ratios of *ccmC* in *P. anserina*, *ccmFn* in *R. chinensis* and *R. rugosa* versus *P. pedunculata* were greater than 1(1.02956, 1.12327, and 1.05751, respectively), inferring positive selection. In the case of *nad2* from the *P. kanzakura* and *P.yedoensis* mitogenome, the Ka/Ks ratios were close to 1 (0.99014), suggesting a tendency of neutral selection. Additionally, 68 pairwise Ka/Ks values were 0 and 120 were pairwise with non-applicable (NA) Ka/Ks values. The highest average Ka/Ks ratios were observed for cytochrome c biogenesis genes (0.62132). Meanwhile, the lowest values of average Ka/Ks ratios were noted for *atp9*(0.0539), *nad3*(0.09952) and *nad9*(0.06257). In particular, the maximum substitution ratios of *atp9*, *atp1*, and *nad9* were as low as 0.235319, 0.325493, and 0.326979, respectively. The low Ka/Ks values suggested that these genes may have been highly conserved during the evolution of the *P. pedunculata* mitogenome.

### 3.5 RNA editing sites prediction

**Figure 6:**
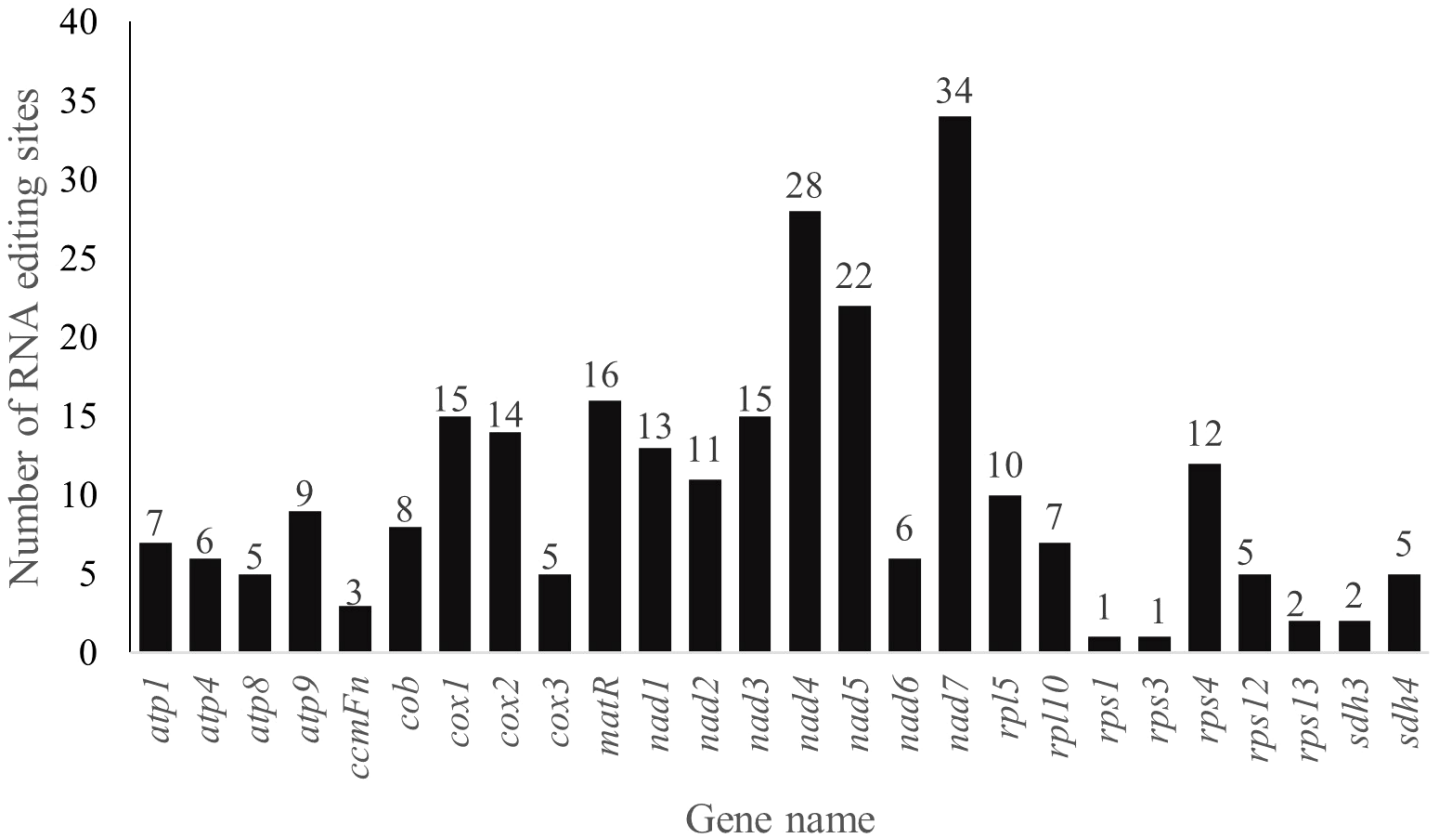
The distribution of RNA editing sites in the mt PCGs of *P. pedunculata*.

RNA editing events, especially the C-to-U editing sites, are enriched in plant mitogenomes. A total of 262 RNA editing sites in 26 analyzed PCGs of *P. pedunculata* mitogenome were identified among which 249 sites exhibiting C-to-U RNA editing (**Figure 6**; **Supplementary Table S9**). Most of the predicted RNA editing events occurred at the first (76, 29.01%) or second (173, 66.03%) positions of the codons, only 13 were found in the third positions (4.96%). The largest number of RNA editing sites was detected in the NADH dehydrogenase genes (129), among them *nad7* (34) maintained the most editing sites in all mitochondrial genes, then followed by *nad4* (28). There is only one editing site were predicted in *rps1* and *rps3*, respectively. No potential RNA editing sites were identified in *atp6*, *ccmB*, *ccmC*, *ccmFc*, *mttB*, *nad4L*, *nad9*, *rps7 and rps13*, no RNA editing sites were found in two assumed pseudogenes (*rpl16*, *rps14*), either. The majority of RNA editing sites (247) were non-synonymous variations, synonymous editing sites were merely (15) found. A total of 31 types of amino acid conversion were identified at these RNA editing sites (**Supplementary Table S9**), including two special sites in *atp9* and *nad7*, which convert to termination codons. The most frequently occurred amino acid changes among all of the identified mutations were histidine (H) to leucine (L) and serine (S) to leucine (L) change, with frequency of 61 times (23.28%) and 55 times (20.99%), respectively. Besides, *nad1* was initiated with ACG as its start codon (**Supplementary Table S7**), implying an alteration caused by RNA editing event.

### 3.6 Chloroplast-derived mitogenomic sequences

**Figure 7:**
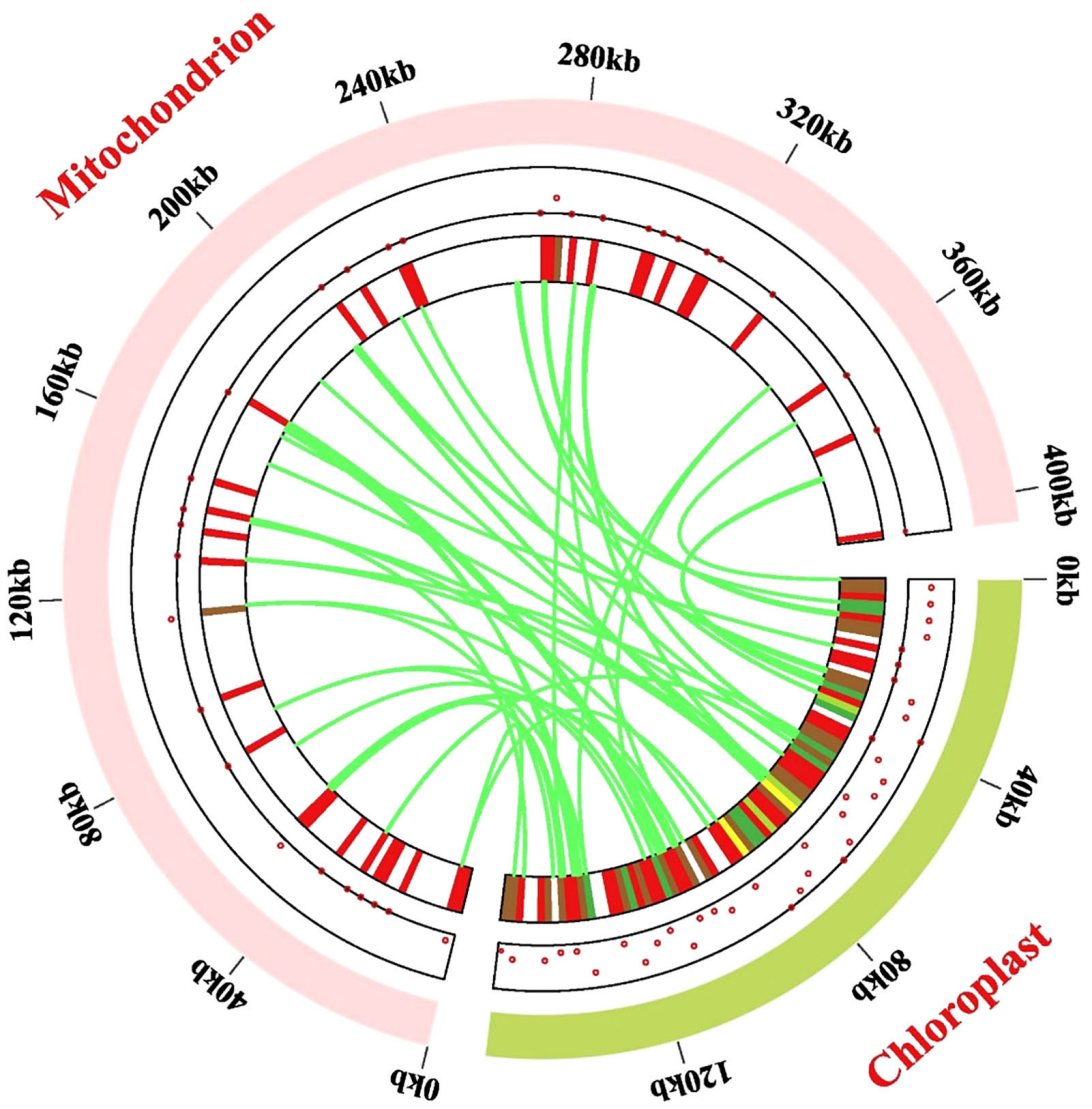
Gene transfer events between the chloroplast and mitochondrial genome. Dots and heat maps inside the two chromosomes demonstrate where the migrated genes are located. The light-green circular segment represents the chloroplast genome, and the pink circular segments depict the mitogenome. The lawn-green lines in the circle portray the routes of chloroplast-like sequences inserted from the cp genome into the mitogenome.

The mitogenome (405, 855 bp) of *P. pedunculata* was approximately 2.57 times larger than the chloroplast genome (157,830 bp), so the distribution of mitochondrial genes in *P. pedunculata* was relatively sparse compared to that of chloroplast genes (**Figure 7**). In this study, 56 chloroplast-like fragments that might have undergone gene transfer were identified in the mitogenome, based on sequence similarity between the chloroplast and mitochondrial genomes of *P. pedunculata* (**Figure 7**; **Supplementary Table S10**). These inserted fragments, ranging from 30 to 863 bp, were distributed on the mitochondrial genome, with a total length of 11,582 bp, which comprised 2.85% of the complete mitogenome. The longest sequence (863 bp) was transferred from *rrn16S* and *rrn16S-2* in the cp genome to *rrn18* in the mitogenome. These migrated sequences were mainly located in the IGS (19) or rRNA genes (20) of the mitogenome. There are 22 *rrn16S* and *rrn23S* sequences of the *P. pedunculata* chloroplast genome that were inserted into the mitogenome and were mostly transferred into *rrn18* or *rrn26*, except for two fragments that were transferred to IGS regions. Among the remaining chloroplast-like sequences, nine fragments were located on tRNA genes, three on core genes (*atp1*, *ccmC*), and two on the ribosomal protein *rsp12*. Some transferred fragments were intact genes (*atp1, rrn18, rrn26, rps12, ccmC, trnD-GTC, trnH-GTG, trnN-GTT, trnF-GAA, trnP-TGG, trnQ-TTG*), whereas others were partial sequences (*trnW-CCA, trnP-TGG-2, trnN-GTT, trnM-CAT-3*).

Migration may occur from gene to gene, from gene to IGS, from IGS to IGS, or from gene to introns/exons. Our findings showed that 22 rRNA sequences were transferred from chloroplasts to the mitochondria, mostly maintaining the function of ribosomal RNAs; whilst only two were inserted into the IGS region and became nonfunctional. Most sequences from the encoded genes of the chloroplasts lost their function and relocated to the IGS of the mitochondria. In contrast, *atpA* from the chloroplasts was transformed into *atp1* in the mitochondria. Five segments that immigrated from the IGS remained in the IGS regions. The transferred sequences of 14 tRNA genes were shared between the chloroplast and mitochondrial genomes, accounting for 25% of the total transferred sequences. Relocated sequences were also observed in the intron and exon regions of some genes.

### 3.7 Phylogenetic analysis

**Figure 8:**
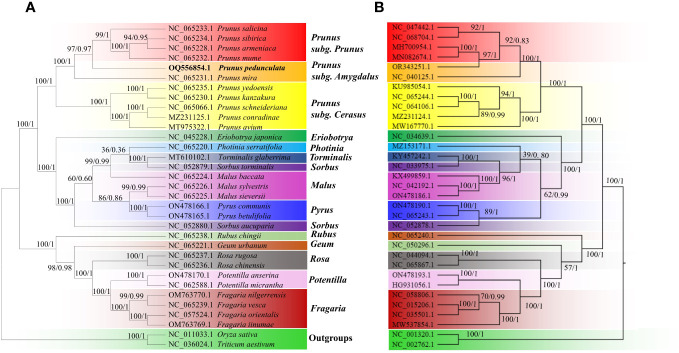
The phylogenetic relationships of *P. pedunculata* compared with that of 30 Rosaceae species. **(A)** Phylogenetic analysis of mitogenomes based on 20 shared protein-coding genes from the mitogenome; **(B)** phylogenetic analysis of cp genomes based on 72 shared protein-coding genes. *Oryza sativa* and *Triticum aestivum* were selected as outgroups. The ML bootstrap support values/BI posterior probabilities are shown for each node. Colored blocks indicate the subtype that the specific species belongs to.

Phylogenetic trees between *P. pedunculata* and 30 other Rosaceae species were constructed using the ML and BI methods, based on 20 shared mitochondrial PCGs and 72 shared chloroplast PCGs, respectively. *O. sativa* and *T. aestivum* functioned as outgroups. (**Figure 8**; **Supplementary Table S1**). Both ML and BI analyses indicated that most branches of the phylogenetic tree had high support values and the topology presented high consistency. The evolution relationships of both chloroplast and mitochondrial genomes among all taxa were separated into three clades, as deduced from the phylogenetic trees. The first large clade consisted of 11 *Prunus* species, which were further clustered into three secondary clades. *P. armeniaca*, *P. sibirica*, *P. mume*, and *P. salicina* form the *Prunus* subclade. As is shown in **Figure 8**, *P. pedunculata* was settled as a single monophyletic branch and group into the *Prunus* subgenus *Amygdalus* clade together with *P. mira* in both organelle genome trees. The subgenus *Cerasus*, including *P. avium, P. conradinae*, *P. schneideriana, P. yedoensis*, and *P. kanzakura* constitutes the third subclade of the *Prunus* genus. These results are consistent with the classification taxonomy.

Ten other ligneous species of Rosaceae, comprising species from the genera *Eriobotrya, Sorbus, Torminalis, Pyrus, Photinia*, and *Malus*, formed Clade 2, among which *E. japonica* and *S. aucuparia* formed distinct subclades, the rest of the species in the group clustered into another subclade in the mitogenome tree, whereas *S. aucuparia* was more related to *Pyrus* in the cp genome tree. Species from the genera *Fragaria*, *Rosa* and *Potentilla* comprised Clade 3. Most species in Clade 3 were herbaceous plants, in addition to three shrub species: *R. chingii*, *R. rugosa*, and *R. chinensis*. *R. chingii* and *G. urbanum* accessions diverged into two independent subclades with different genetic distance in the mitogenome tree and cp genome tree, though they are separated from the other relatives in this group.

## 4 Discussion

### 4.1 Genomic features of the *P. pedunculata* mitogenome

Mitochondria have more complex genomes in plants than those of animals, owing to variation and repeated sequences. Due to the complexity of plant mitogenomes, extensive research has been focused on plastids, leaving multifarious mitogenomes to be investigated (Zardoya, 2020). Each of the 60 Rosaceae mitogenomes deposited in NCBI database was assembled into cyclic structure with remarkable variation in size, ranging from 270, 143bp (*Rosa* hybrid cultivar: OQ628291.1) to 535,727bp (*P. mume*: NC_065232.1). The genome size within the *Prunus* species varies slightly (**Supplementary Table S1**), among which *P. pedunculata* mitogenome (405, 855bp) was the smallest. In this survey, the complete mitogenome of *P. pedunculata* was thoroughly characterized and assembled into a typical circular structure. The GC contents of Rosaceae mitogenomes are relatively static, ranging from 43.31% in *R. chingii* to 45.62% in *P. avium* (Sun et al., 2022). Interestingly, *P. pedunculata* exhibited the highest percentage of GC base (45.63%). The corresponding chloroplast genome sequence of *P. pedunculata* was found to be 157,830 bp long, with a GC content of 36.8%.

Plant mitogenomes contain highly conserved genes, relatively low gene densities, abundant non-coding sequences, and RNA editing occurrences (Niu et al., 2023). The high account of non-coding regions (84.82%) of the *P. pedunculata* mitogenome might be a consequence of sequence duplication during evolution (Ma et al., 2022). Mitochondrial DNA (mtDNA) encodes tRNAs, rRNAs, and a dynamic number of ribosomal proteins (Kubo and Newton, 2008). A total of 34 PCGs were identified in the *P. pedunculata* mitogenome, which is higher than that of most mitogenomes of Rosaceae species, aside from *P. salicina*, which has 39 PCGs in its mitogenome. The high content of PCGs indicated that fewer original mitochondrial genes had transfer into nuclear region during the evolutionary history of *P. pedunculata*. No gene loss events were observed in PCGs of the *P. pedunculata* mitogenome. Thirty-five genes were detected in most *Prunus* mitogenomes, except for *rpl16* which was found only in the *P.avium* cultivar Glory, the *P. avium* cultivar Staccato, *P. tenella*, *P. armeniaca*, and *P. salicina* (Sun et al., 2022). Additionally, similar with *O. sativa* (Kazama and Toriyama), the *P. pedunculata* mitogenome contains two duplicated loci of the *atp6* gene, one of them was assumed to be a pseudo-copy. Most Rosaceae species contain three ribosomal RNAs in their mitogenomes, except some species that possess four rRNAs (*G. urbanum*, *M. domestica* cultivar Gala, *M. domestica* cultivar Yantai fuji 8, *M. sylvestris* and *P. serratifolia*), and *S. aucuparia* has two rRNAs (Sun et al., 2022). In accordance with the known mitogenomes of *Prunus*, the mitogenome of *P. pedunculata* contains three rRNAs (*rrn18, rrn26*, and *rrn5*). In general, transfer RNAs in the mitogenome of angiosperm do not form a complete set (Michaud et al., 2011). Eight tRNAs *(trnP-AGG*, *trnP-GGG*, *trnR-TCT*, *trnS-ACT*, *trnS-GGA*, *trnT-GGT*, *trnV-GAC*, and *trnI-GAT*) were absent in the mitogenomes of *Prunus*, including *P. pedunculata*. Specifically, there are two copies of *trnC-GCA* in *Prunus* species and *trnN-ATT* appears solely in *Prunus* mitogenomes. Three copies of *trnM-CAT*, two copies of *trnP-TGG*, and *trnY-GTA* have been identified in most *Prunus* species. Moreover, t*rnI-TAT* and *trnL-TAA* were absent from *P. pedunculata*, *P. schneideriana*, *P. conradinae*, and *P. tenella*, whereas *trnK-TTT* and *trnG-GCC* were present in these four species, differs greatly from other *Prunus* species (Sun et al., 2022). As for the intron-containing tRNAs (*trnN-ATT*, *trnT-TGT* and *trnY-GTA*) in *P. pedunculata* mitogenome, splicing their introns is a critical step of tRNA maturation (Hayne et al., 2022).

Repetitive sequences, especially abundant SSRs (Freitas et al., 2022), increase the mitogenome size, generation of multiple copies of genes, genome diversification, and structural variations (Fang et al., 2021). SSRs are characterized by cross-species transfer and high polymorphism, which allow them to be applied as molecular markers in phylogenetic analyses (Rossetto et al., 2002). In our study, 112 SSRs of *P. pedunculata* mitogenome distributed across different genomic regions: ORFs, introns, exons, or intergenic regions (IGS). The most abundant type of SSR in the mtDNA was mononucleotides (40.18%), consistent with a previous study (Kuntal and Sharma, 2011). However, the second-most common SSR in *P. pedunculata* mitogenome was tetranucleotide repeats (35.71%), rather than dinucleotide repeats. A near-universal A/T bias (Smith and Keeling, 2015) was also observed in *P. pedunculata* mitogenome, these SSRs were composed of motifs rich in A and T, which agrees with previous observations (Kuntal and Sharma, 2011), and corroborated the correlation between AT content of the complete mitogenome and SSRs (Freitas et al., 2022).

### 4.2 The genomic variation of *P. pedunculata* mitogenome

The non-synonymous and synonymous substitution ratios (Ka/Ks) have a complex relationship with biological functions of PCGs, and reflect gene selective pressures, thus contributing to our understanding of the evolutionary dynamics of PCGs among related species (Wang et al., 2010). In the current study, for the cytochrome c biogenesis genes *ccmC* and *ccmFn* that underwent positive selection (Ka/Ks>1), non-synonymous variations were favored because of functional adaptation or useful mutations. The average Ka/Ks value was the largest (0.62132) for cytochrome c biogenesis genes. Stabilizing selection (Ka/Ks>1) have been reported for other plants mitochondrial genes (Bi et al., 2020; Cheng et al., 2021; Yu et al., 2022). In contrast, genes that with Ka/Ks ratios below 1 may be highly conserved and have undergone negative selection during evolution (Greilhuber et al., 2012). Nonsynonymous variations are likely to be disadvantageous, therefore, genes with crucial functions subjected to stabilizing selection are partial to having lower Ka/Ks values. The lowest average values were observed for genes related to NADH dehydrogenase (0.18653). Because of constraint on protein function, synonymous substitutions are more common in most mitochondrial PCGs of *P. pedunculata* that dominated by purifying selection, which concordant with the fact that PCGs in the mitogenome are conserved across green plants (Mower et al., 2007; Cheng et al., 2021). With the Ka/Ks value approximately equal to 1, the protein function of *nad2* might not constrain evolutionary changes. These results implied that the majority of genes were subjected to purifying selection. Thus, further research of the gene selection and evolution of *Prunus* species is still required.

Selection events for biased codon usage and recognition motifs for RNA editing sites occur in angiosperm mitogenomes (Liu and B., 2005). Deaminated cytosines in RNA transcripts become uridines at RNA editing sites in numerous mitochondrial DNA-encoded genes (Morley and Nielsen, 2017; Hao et al., 2021). According to the RSCU analysis, the most unique PCGs encoded by the *P. pedunculata* mitogenome contained the classical start codon ATG, in accordance with the allocation of amino acid compositions in other plant mitogenomes (Sloan et al., 2018b). Nevertheless, *nad1* starts with ACG, presumably as a consequence of C-to-U editing at the second site. The A/T bias in the third codon position of PCGs with higher RSCU values (>1) indicates that A/T (U) stews across the mitochondrial genome of *P. pedunculata*, which may be a consequence of the A/T mutation bias common in plant mitogenomes (Smith and Keeling, 2015; Bi et al., 2020). CGA acts as a stop codon for *ccmFc, atp9*, and *sdh4*; however, analogous observations have rarely been reported. The *cox1* gene of many lepidopteran species uses CGA as a start codon (Wu et al., 2020a; Jiang et al., 2021) rather than a stop codon. CAA acts as a stop codon for *atp6* in the *P. pedunculata* mitogenome. Similar results have been reported for *atp9* in wild carrots (Mandel et al., 2012).

It is believed that RNA editing events plays a pivotal role in the plant development regulation and stress resistance (Tang and Luo, 2018). In general, plant mitochondria contain more RNA editing sits than chloroplast (Small et al., 2020). We have predicted 262 RNA editing sites in *P. pedunculata* mitogenome, which is lower in comparison to other angiosperms (Giegé and Brennicke, 1999; Sloan et al., 2010; Richardson et al., 2013; Shan et al., 2023). Most RNA editing events in plant organelles arise from the site-specific C-to-U conversion (Small et al., 2020). Similar results were found in the current research. Particularly, 3 reverse changes (U-to-C conversion) were also been identified, which is more likely enriched in basal plants (Chen et al., 2023). In addition, G-to-A, G-to-U, A-to-G, A-to-U, U-to-A and C-to-A conversion types were also observed once and G-to-C changes were found twice among all RNA editing sites. RNA editing events may trigger the variation of amino acids and change the start or stop codons of PCGs. There are editing sites being transformed to stop codons in *atp9* and *nad7*. ACG was used as a start codon in *nad1* gene of *P. pedunculata* mitochondria directly, possibly due to a C-to-U conversion at the second site without editing in mitogenome (Zandueta-Criado and Bock, 2004). RNA editing events were reported existing in the initiation codon of *nad1*in other plants (Li et al., 2018; Fang et al., 2021; Wee et al., 2022). Consistent to most plants, there is few RNA editing events occur in rRNA, tRNA and introns (Giegé and Brennicke, 1999). In view of previous studies, RNA editing sites barely exist at the third codon position (Bi et al., 2020; Ma et al., 2022), largely because of the limitations of the methodology. Therefore, further research on the precisely predictive methods is still required.

Intergenomic gene transfer between organellar genomes has been an important phenomenon throughout the long-term evolution of higher plants (Sloan and Wu, 2014). As an important type of intracellular gene transfer (IGT) (Wang et al., 2018d) in the mitochondria, the mechanism of transfer events remains challenging because most transferred DNA sequences are located in non-coding regions. Plant mitogenomes maintain massive mitochondrial plastid fragments (MTPTs) (Lai et al., 2022). The proportion of plastid-derived fragments in mitogenomes varies from 0.44% (*P. serratifolia*) to 16.34% (*R. chingii*) in the family Rosaceae (Sun et al., 2022). The integration rate of chloroplast-derived mitogenomic sequences in *P. pedunculata* was 2.85%, which was much higher than that in other *Prunus* species (less than 1.16%). Similarly, the total length of the transferred sequences in the *P. pedunculata* mitogenome was 11,582 bp, which was the longest among the *Prunus* species. The species with the highest total length of plastid-derived sequences in the Rosaceae family is *R. chingii* (77,163 bp), which also comprises the highest proportion (16.34%) (Sun et al., 2022). Mostly, chloroplast-derived sequences are non-functional (Zhang et al., 2023). Remarkably, fragments from the exons of two genes (*rps12, rps12-2*) in the chloroplast genome of *P. pedunculata* changed to functional *rps12* after transfer. Conversely, as a consequence of gene transfer events, the original functional gene sequence of *ndhF* was relocated to the exon region of *nad5*. The *rrn16*-*rrn23* regions were used to construct transformation vectors, indicating that the *rrn16*-*rrn23* chloroplast region might affect transformation efficiency (Lopez-Ochoa et al., 2015). The lost tRNAs in mitogenomes can be replaced by tRNAs inserted into other organelles (Sloan et al., 2010). The transfer of tRNA gene sequences from chloroplasts to mitochondrial genomes are common in plants (Bergthorsson et al., 2003). Inserted tRNAs accounted for 25% of the total transferred sequences in the *P. pedunculata* mitogenome, which contradicts the hypothesis that most transferred genes are tRNA genes (Chang et al., 2013).

Polyploidy of *P. pedunculata* leads to difficulties in the evolutionary analysis of genome sequences and phenotypes (Wang et al., 2020a). Phylogenetic inference of *P. pedunculata* has been controversial. According to previous publications, *P. pedunculata* was placed outside the monophyletic subgenus *Amygdalus* based on molecular data. In terms of morphological classification, it is clustered as a sister clade to peach (Yazbek and Oh, 2013). Previous studies have suggested that *P. pedunculata* is closely associated with *P. tomentosa* and *P. triloba* (Wang et al., 2018a; Duan et al., 2020; Wang et al., 2020a; Wang et al., 2021a). Nevertheless, there were no complete mitochondrial genome data for these two species available for phylogenetic analysis In this study, within the *Prunus* clade, *P. pedunculata* was divided into an independent branch, suggesting that the mitogenome divergence process produced distinctive maternal lines of *P. pedunculata*. The analysis provided strong evidence of the phylogenetic affinities of *P. pedunculata* from the perspective of 20 conserved PCGs in the mitogenome and provided a better understanding of the phylogenetic relationships among Rosaceae species. Nevertheless, additional investigations of more accessions of *Prunus* species and more molecular data are required to comprehensively understand the phylogeny of *Prunus* (Pervaiz et al., 2015).

## 5 Conclusions

In this study, the complete mitochondrial genome of *P. pedunculata* was assembled and characterized. Extensive analyses have been conducted on the mitogenome features. The *P. pedunculata* mitogenome is a circular molecule with a total length of 405,855 bp, which encodes 62 genes, including 34 PCGs, three ribosomal RNAs, and 19 transfer RNAs. The mitogenome contained 112 SSRs, 15 tandem repeats, and 50 interspersed repetitive sequences with a total repeat length of 11, 793 bp. The codon usage of PCGs, Ka/Ks ratios, RNA editing and gene transfer events in the *P. pedunculata* mitogenome were thoroughly evaluated. This phylogenetic relationship analysis was supported by mitogenome information of 30 other taxa of the Rosaceae family. In summary, understanding the mitochondrial genome characteristics of *P. pedunculata* is of great importance to promote the understanding of the evolution of the genetic background and provide a basis for genetic breeding of *Prunus*.

## Data availability statement

The datasets presented in this study can be found in online repositories. The names of the repository/repositories and accession number(s) can be found in the article/**Supplementary Material**. The raw data were deposited at NCBI SRA database https://www.ncbi.nlm.nih.gov/sra; with the accession number: SRR25636420.

## Author contributions

ZW: Formal analysis, Methodology, Writing – review & editing. QL: Formal analysis, Investigation, Validation, Writing – original draft. CT: Resources, Writing – review & editing. YY: Investigation, Writing – review & editing. LL: Validation, Writing – review & editing. YF: Formal analysis, Writing – review & editing. ZL: Project administration, Supervision, Writing – review & editing.
