# Supplementary figures and images for: Complete mitochondrial genome of the endangered Prunus pedunculata (Prunoideae, Rosaceae) in China: characterization and phylogenetic analysis
Source: Front Plant Sci. 2023 Dec 8;14:1266797. doi: 10.3389/fpls.2023.1266797 (PMC10753190; doi:10.3389/fpls.2023.1266797)

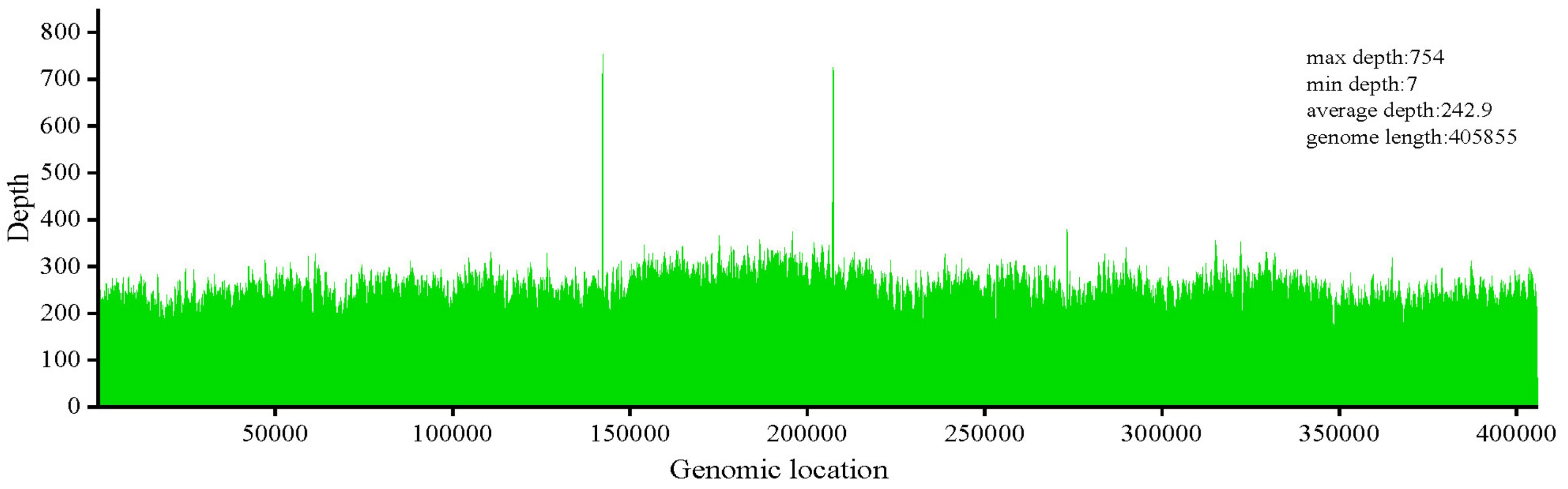

Supplement: Supplementary file 1 [file Image_1.jpeg]
